# Cost-effectiveness of Housing First Intervention With Intensive Case Management Compared With Treatment as Usual for Homeless Adults With Mental Illness: Secondary Analysis of a Randomized Clinical Trial

**DOI:** 10.1001/jamanetworkopen.2019.9782

**Published:** 2019-08-21

**Authors:** Eric A. Latimer, Daniel Rabouin, Zhirong Cao, Angela Ly, Guido Powell, Carol E. Adair, Jitender Sareen, Julian M. Somers, Vicky Stergiopoulos, Andrew D. Pinto, Erica E. M. Moodie, Scott R. Veldhuizen

**Affiliations:** 1 Department of Psychiatry, McGill University, Montreal, Quebec, Canada; 2 Douglas Research Centre, Montreal, Quebec, Canada; 3 Montreal West Island Integrated University Health and Social Services Centre, Montreal, Quebec, Canada; 4 Department of Epidemiology, Biostatistics, and Occupational Health, McGill University, Montreal, Quebec, Canada; 5 Department of Psychiatry, University of Calgary, Calgary, Alberta, Canada; 6 Department of Community Health Sciences, University of Calgary, Calgary, Alberta, Canada; 7 Department of Psychiatry, Faculty of Medicine, University of Manitoba, Winnipeg, Manitoba, Canada; 8 Faculty of Health Sciences, Simon Fraser University, Vancouver, British Columbia, Canada; 9 Department of Psychiatry, University of Toronto, Toronto, Ontario, Canada; 10 Centre for Addiction and Mental Health, University of Toronto, Toronto, Ontario, Canada; 11 Department of Family and Community Medicine, Faculty of Medicine, University of Toronto, Toronto, Ontario, Canada; 12 MAP Centre for Urban Health Solutions, Li Ka Shing Knowledge Institute, St Michael’s Hospital, Toronto, Ontario, Canada

## Abstract

**Importance:**

In the At Home/Chez Soi trial for homeless individuals with mental illness, the scattered-site Housing First (HF) with Intensive Case Management (ICM) intervention proved more effective than treatment as usual (TAU).

**Objective:**

To evaluate the cost-effectiveness of the HF plus ICM intervention compared with TAU.

**Design, Setting, and Participants:**

This is an economic evaluation study of data from the At Home/Chez Soi randomized clinical trial. From October 2009 through July 2011, 1198 individuals were randomized to the intervention (n = 689) or TAU (n = 509) and followed up for as long as 24 months. Participants were recruited in the Canadian cities of Vancouver, Winnipeg, Toronto, and Montreal. Participants with a current mental disorder who were homeless and had a moderate level of need were included. Data were analyzed from 2013 through 2019, per protocol.

**Interventions:**

Scattered-site HF (using rent supplements) with off-site ICM services was compared with usual housing and support services in each city.

**Main Outcomes and Measures:**

The analysis was performed from the perspective of society, with days of stable housing as the outcome. Service use was ascertained using questionnaires. Unit costs were estimated in 2016 Canadian dollars.

**Results:**

Of 1198 randomized individuals, 795 (66.4%) were men and 696 (58.1%) were aged 30 to 49 years. Almost all (1160 participants, including 677 in the HF group and 483 in the TAU group) contributed data to the economic analysis. Days of stable housing were higher by 140.34 days (95% CI, 128.14-153.31 days) in the HF group. The intervention cost $14 496 per person per year; reductions in costs of other services brought the net cost down by 46% to $7868 (95% CI, $4409-$11 405). The incremental cost-effectiveness ratio was $56.08 (95% CI, $29.55-$84.78) per additional day of stable housing. In sensitivity analyses, adjusting for baseline differences using a regression-based method, without altering the discount rate, caused the largest change in the incremental cost-effectiveness ratio with an increase to $60.18 (95% CI, $35.27-$86.95). At $67 per day of stable housing, there was an 80% chance that HF was cost-effective compared with TAU. The cost-effectiveness of HF appeared to be similar for all participants, although possibly less for those with a higher number of previous psychiatric hospitalizations.

**Conclusions and Relevance:**

In this study, the cost per additional day of stable housing was similar to that of many interventions for homeless individuals. Based on these results, expanding access to HF with ICM appears to be warranted from an economic standpoint.

**Trial Registration:**

isrctn.org Identifier: ISRCTN42520374

## Key Points

**Question:**

Is a Housing First intervention with Intensive Case Management for homeless people with mental illness cost-effective compared with treatment as usual?

**Findings:**

In this economic evaluation study of data from the At Home/Chez Soi randomized clinical trial with 1198 initially homeless participants, the incremental cost-effectiveness ratio was $56.08 per additional day of stable housing. At $67 per day of stable housing, there was an 80% chance that the Housing First intervention with Intensive Case Management was cost-effective compared with treatment as usual.

**Meaning:**

Expanding access to Housing First with Intensive Case Management appears to be warranted from an economic point of view.

## Teaser

This economic evaluation study investigates the cost-effectiveness of the Housing First intervention with Intensive Case Management compared with treatment as usual among homeless adults with mental illness.

## Introduction

A significant proportion of homeless individuals experience mental illness.^1^ Housing First (HF), which provides immediate access to subsidized housing together with support services, has proven to be the most effective approach at helping such individuals access and maintain permanent housing.^2,3^ Previous analyses,^4^ using mostly before-and-after comparisons or quasi-experimental designs, have reported significant cost offsets associated with the provision of HF. To our knowledge, only 1 cost-effectiveness analysis,^5^ conducted alongside a randomized clinical trial, has been published.

The multiple-site At Home/Chez Soi trial compared outcomes of the scattered-site variant of HF, in which participants receive income-related rent supplements for private market apartments of their choice, with those of treatment as usual (TAU). The trial tested, in parallel, HF with Assertive Community Treatment for people who had more severe mental illness and functional difficulties and HF with Intensive Case Management (ICM) for those whose needs were less acute.^6^ Summary results of cost analyses, but not cost-effectiveness analyses, were included in the main trial reports.^7,8^ Herein we report on the cost-effectiveness of HF with ICM compared with TAU.

## Methods

The cost-effectiveness analysis was performed in conformity with the published protocol of the At Home/Chez Soi study.^6^ The analysis followed the Consolidated Health Economic Evaluation Reporting Standards (CHEERS) reporting guideline.^9^ The analysis began in 2013 and underwent successive refinements until 2019. The study protocol is available elsewhere.^6^ The trial was conducted in the Canadian cities of Vancouver, British Columbia; Winnipeg, Manitoba; Toronto, Ontario; Montreal, Québec; and Moncton, New Brunswick. Ethics approval was obtained from the local ethics review board at each data-collection site and from the Centre for Addiction and Mental Health, where the coordinating center was based.^6^ Participants provided written informed consent after the screening interview.

### Participants

Details on sample recruitment are available elsewhere.^6,7^ Briefly, potential participants were referred from various sources or found through street outreach. Individuals were eligible if they were adults with legal status in their province of residence; had at least 1 of 6 current mental disorders, including psychotic disorder, major depressive disorder, or posttraumatic stress disorder; and were absolutely homeless or precariously housed with previous episodes of absolute homelessness. Individuals who were currently receiving services from an Assertive Community Treatment or ICM team (similar to what the experimental interventions offered, minus the access to rent supplements and dedicated housing staff) were not eligible.

### Initial Assessment and Randomization

Using data collected online during the baseline interview, a computerized algorithm classified individuals as high or moderate need. To be classified as high need, individuals needed to (1) have a lower level of functioning (score of ≤62 on the Multnomah Community Ability Scale)^10^; (2) have a diagnosis of current psychotic disorder or bipolar disorder; and (3) meet at least 1 of the following criteria: 2 or more hospitalizations for mental illness during a 1-year period within the previous 5 years; comorbid substance use; or 1 or more arrests or incarcerations in the past 6 months. Others were classified as moderate need. (In Moncton, high-need and moderate-need individuals were classified as high need, because of the relatively small pool of individuals eligible for the study.^6^ Accordingly, data from Moncton are not included in this analysis.) Moderate-need individuals were randomized to receive HF plus ICM or other services normally available to them (TAU).^7^ An adaptive randomization algorithm^11^ with allocation concealment was used. From October 2009 through June 2011, 1198 moderate-need individuals were recruited.

### Interventions

Participants in the HF plus ICM group received recovery-oriented supports from an ICM team with about 17 participants per case manager. Each ICM team worked in collaboration with housing specialists, also paid by the project, to help participants find housing of their choice, usually an apartment on the private rental market, and respond to housing issues as they arose. Participants were required to pay 25% or 30% of their income toward the rent, depending on whether it covered heating costs. The project paid the remainder of the rent, with this supplement ranging from a mean of $375 in Montreal to $600 in Vancouver. Periodic evaluation of the fidelity of the interventions to the program model, combined with feedback and ongoing coaching, aided standardization of the interventions across sites.^12^

Although their intervention did not include HF, participants assigned to the TAU group had access to substantial supports, especially in the larger cities.^13^ These supports included emergency response services, such as shelters and hospital emergency departments, and some rehabilitative services, such as drug and alcohol rehabilitation centers and transitional housing. A small number of participants also were able to access ICM or Assertive Community Treatment services after they were recruited into the study.

### Data Collection

Study participants were followed up for as long as 24 months. At baseline, interviewers used the Mini International Neuropsychiatric Interview^14^ and other sources such as medical records to ascertain diagnosis, assess whether abuse or dependence of alcohol or other substances was present, and document homelessness, hospitalization, and arrest history, among others. At baseline and every 6 months thereafter, a battery of standardized questionnaires was administered. The Multnomah Community Ability Scale, the only interviewer-rated measure, was completed at the end of the interview. Measures also included 3 questionnaires adapted for this study and designed to assess use of services.^6,13^ Participants completed the Health Services and Justice Services Use questionnaire at baseline and every 6 months thereafter. The Health Services and Justice Services Use questionnaire documented all non–overnight health- and justice-related services.^6^ The Residential Time-Line Follow-Back instrument was administered every 3 months starting 3 months after baseline and asked participants where they had spent every night since the previous interview (or since 3 months before baseline).^6^ To enable estimation of costs associated with service use, the Residential Time-Line Follow-Back instrument allowed coding of simultaneous places of residence; for example, if a participant had a subsidized apartment and was hospitalized, costs were associated with both places concurrently. Finally, the Vocational Time-Line Follow-Back questionnaire asked about income received month by month and any regular or casual work obtained during the previous 3 months.^6^ Due to the nature of the intervention and the inclusion of measures on service use and housing, participants and interviewers could not be blinded.

### Choice of Outcome Measure

Days of stable housing (as assessed by the Residential Time-Line Follow-Back instrument) served as the outcome measure. Places where people stayed were classified as stable (own apartment, social housing, or staying with one’s family if this could be maintained for ≥6 months) or unstable. Thus, all other housing situations, for this purpose, were deemed unstable.

### Perspective of the Economic Analysis

As discussed in detail elsewhere,^13^ cost elements were collected and analyzed from the perspective of society.^15^ We modified this perspective slightly, following Weisbrod et al,^16^ in that we included social assistance and disability benefits as costs. This modified societal perspective may be viewed as consistent with a social cost impact analysis.^17,18^

### Calculation of Costs per Individual

We calculated many unit costs at a high level of specificity, distinguishing, for example, among supportive housing providers with different staffing levels. Whenever possible, we used financial statements and activity reports to estimate a fully allocated average cost of each service.^19^ The unit costs that we used and the methods that we used to derive them have been published.^13^

Unit costs for the intervention were based on reported expenses of each clinical team and housing provider. Program expenses were distributed among participants based on their own time receiving services from their clinical team, as estimated using the Health Services and Justice Services Use questionnaire, and on the number of nights that they had a subsidized apartment or housing unit provided by the project.

All unit costs were originally in 2011 Canadian dollars or adjusted to 2011 Canadian dollars. For this analysis, we used the city-specific Consumer Price Index to convert costs into 2016 Canadian dollars.^20^ We calculated costs per individual by multiplying frequencies by the corresponding unit cost, including the intervention cost for experimental group participants, adding to that social assistance and other contributions by society to their income, and finally subtracting income earned.^13^

### Discounting

For each participant, costs as well as days of stable housing were estimated for a 2-year period. Costs and days of stable housing in the second year were discounted at a 3% rate, a common rate for a base case analysis.^19^

### Statistical Analysis

All analyses used multiple imputation with chained equations (20 imputations) to account for missing data.^21^ Mean costs per year after randomization, aggregated across sites but grouped into different categories,^13^ were compared between the HF and TAU groups at baseline and during each of the 2 years after baseline. Mean total costs per year were then compared site-by-site between the HF plus ICM and TAU groups.

Confidence intervals for incremental cost-effectiveness ratios were computed via bootstrapping, with 500 bootstrap resamples.^21,22^ We plotted the bootstrap resamples on the cost-effectiveness plane.

We used the net-benefit approach to describe further the effect of sampling uncertainty.^19^ The intervention is deemed cost-effective if λμ_ΔE_ – μ_ΔC_ > 0, where λ is the threshold ratio (in dollars per additional day of stable housing) above which the decision-maker no longer finds the intervention cost-effective; μ_ΔE_, the mean difference in effectiveness between the 2 groups; and μ_ΔC_, the mean difference in costs. Using the bootstrap resamples, we plotted the cost-effectiveness acceptability curve, showing the estimated probability that the intervention is cost-effective as a function of λ.

We then regressed, using values of λ ranging from $0 to $100, each individual’s net monetary benefit on several variables selected a priori as potentially relevant, including group assignment, site, age, sex, presence of psychotic disorder, Multnomah Community Ability Scale score, duration of longest previous episode of homelessness, and number of hospital days in the year before study entry. Linear regression was used in each case. To evaluate how participant characteristics might mediate the cost-effectiveness of HF,^19,23^ and in the absence of any strong a priori hypotheses about which characteristics might be relevant, we then tested, one by one, interactions between all these variables and the group assignment variable. Interaction terms with 2-sided *P* < .10 were retained for a final model with interactions. Fitted models were checked for misspecification by plotting the residuals against the fitted value of the dependent variable as well as continuous covariates. The Rubin rule was used to derive 95% CIs.^21^ Statistical analyses were performed using Stata, version 15 (StataCorp).

### Sensitivity Analyses

We tested the robustness of the results to the choice of discount rate by using 0% and 5% instead of 3%. We also checked the effects of adjusting for baseline differences in costs using a regression-based method^24^ and performed a 2-way sensitivity analysis on these factors.

## Results

Of 1198 individuals originally randomized (795 [66.4%] male, 390 [32.6%] female, and 13 [1.1%] other; 696 [58.1%] aged 30 to 49 years), 1160 (96.8%) provided usable data for this analysis. eFigure 1 in the Supplement describes the flow of participants into and through the trial and shows the available sample size by group and by site.

Table 1 provides descriptive statistics for the sample at baseline. Values for other variables not used in this analysis have been reported elsewhere.^7^ Their mean (SD) longest period of homelessness was 29.0 (42.6) months (median, 12 months [interquartile range, 5-36 months]).

**Table 1.** Baseline Characteristics of Participants, Stratified by Randomization Group

Characteristic	Study Randomization Group	
	HF (n = 689)	TAU (n = 509)
Age group, y, No. (%)		
<30	118 (17.1)	91 (17.9)
30-49	398 (57.8)	298 (58.4)
≥50	173 (25.1)	120 (23.6)
Sex, No. (%)		
Women	236 (34.3)	154 (30.3)
Men	449 (65.2)	346 (68.0)
Other	4 (0.6)	9 (1.8)
Alcohol abuse or dependence, No. (%)^a^	296 (43.0)	224 (44.0)
Substance abuse or dependence, No. (%)^a^	321 (46.6)	242 (47.5)
Alcohol or substance abuse or dependence, No. (%)^a^	424 (61.5)	321 (63.1)
Hospitalization history, No. (%)^b^	155 (22.5)	129 (25.3)
Arrest history, No. (%)^c^	200 (29.0)	154 (30.3)
Longest period homeless, mo		
Mean (SD)	29.7 (46.4)	28.1 (37.0)
Median (IQR)	12 (6-36)	12 (5-36)
MCAS score^d^		
Mean (SD)	64.7 (6.2)	64.7 (6.2)
Median (IQR)	65.0 (63.0-68.0)	65.0 (63.0-68.0)
Study site, No. (%)		
Montreal, Québec	204 (29.6)	102 (20.0)
Toronto, Ontario	204 (29.6)	174 (34.2)
Winnipeg, Manitoba	181 (26.3)	133 (26.1)
Vancouver, British Columbia	100 (14.5)	100 (19.6)

Abbreviations: HF, Housing First intervention; IQR, interquartile range; MCAS, Multnomah Community Ability Scale; TAU, treatment as usual.

^a^ Evaluated using the Mini International Neuropsychiatric Interview.^14^

^b^ Indicates 2 or more hospitalizations within 1 year during the 5 years before baseline.

^c^ Indicates 1 or more arrests or incarcerations during the 6 months before baseline.

^d^ Scores range from 17 to 85, with higher values indicating better functioning.

Table 2 shows baseline and first- and second-year costs by type of cost for the HF and TAU groups. During the 2-year follow-up period, meaningful cost offsets (mean reductions in costs attributable to the intervention) were observed for shelters (−$2627; 95% CI, −$3232 to −$2079), substance use treatment (−$1148; 95% CI, −$1658 to −$638), supportive housing (−$1861; 95% CI, −$2540 to −$1222), and ambulatory visits (−$2375; 95% CI, −$3226 to −$1523). For other cost categories, the 95% CIs for offsets for other cost categories included zero, or the point estimate was less than $1000. Excluding the intervention cost, the total mean cost offset was −$6629 (95% CI, −$10 199 to −$2969). However, after including the mean cost of the intervention ($14 496, inferred from Table 2), the mean total cost for HF participants exceeded that for TAU by $7868 (95% CI, $4409-$11 405). Thus, 46% of the cost of the intervention was offset. For most services as well as in total, the cost difference was less favorable to HF in the second year than in the first.

**Table 2.** Mean (Unadjusted) Costs per Person per Year by Time and Cost Category^a^

Cost Category	Study Randomization Group, Mean (95% CI), 2016 Can$		
	TAU (n = 483)	HF (n = 677)	Difference (HF − TAU)
Shelters			
Baseline	9595 (8680 to 10 434)	8915 (8204 to 9650)	−679 (−1876 to 425)
First year	5476 (4803 to 6184)	2244 (1940 to 2535)	−3233 (−4037 to −2537)
Second year	2879 (2348 to 3479)	857 (626 to 1084)	−2022 (−2625 to −1404)
Mean of first and second years	4177 (3684 to 4773)	1550 (1320 to 1762)	−2627 (−3232 to −2079)
Substance use treatment			
Baseline	3553 (2580 to 4750)	3320 (2358 to 4335)	−233 (−1692 to 1264)
First year	2400 (1724 to 3149)	986 (733 to 1279)	−1414 (−2190 to −697)
Second year	1666 (1130 to 2123)	785 (561 to 1074)	−881 (−1377 to −304)
Mean of first and second years	2033 (1580 to 2521)	886 (687 to 1105)	−1148 (−1658 to −638)
Supportive housing^b^			
Baseline	2476 (1805 to 3195)	3461 (2823 to 4279)	985 (0 to 2073)
First year	3353 (2727 to 4097)	1517 (1263 to 1812)	−1835 (−2550 to −1137)
Second year	2938 (2243 to 3656)	1052 (706 to 1476)	−1886 (−2691 to −1081)
Mean of first and second years	3145 (2549 to 3769)	1285 (1036 to 1573)	−1861 (−2540 to −1222)
Ambulatory visits			
Baseline	14 920 (12 580 to 17 358)	11 629 (10 146 to 13 586)	−3291 (−6026 to −450)
First year	7572 (6520 to 8663)	4545 (3904 to 5219)	−3028 (−4237 to −1653)
Second year	5708 (4833 to 6661)	3986 (3423 to 4709)	−1722 (−2802 to −587)
Mean of first and second years	6640 (5987 to 7435)	4266 (3809 to 4755)	−2375 (−3226 to −1523)
ED visits			
Baseline	2207 (1870 to 2590)	2092 (1789 to 2444)	−115 (−611 to 377)
First year	1862 (1500 to 2261)	1239 (1077 to 1418)	−623 (−1055 to −237)
Second year	1279 (1033 to 1572)	981 (856 to 1105)	−299 (−641 to −22)
Mean of first and second years	1571 (1325 to 1876)	1110 (995 to 1238)	−461 (−775 to −186)
Hospitalizations (physical)			
Baseline	1621 (624 to 2990)	1567 (612 to 2864)	−54 (−1649 to 1688)
First year	2493 (1533 to 3669)	2025 (1226 to 2943)	−468 (−1906 to 975)
Second year	2786 (1443 to 5106)	2421 (1456 to 3546)	−365 (−2840 to 1376)
Mean of first and second years	2640 (1722 to 4005)	2223 (1586 to 2939)	−417 (−2060 to 799)
Hospitalizations (psychiatric)			
Baseline	4285 (2153 to 6554)	4507 (2660 to 6516)	222 (−2776 to 3450)
First year	3392 (1876 to 5328)	3670 (2442 to 5334)	278 (−2185 to 2579)
Second year	1644 (975 to 2578)	4414 (2714 to 6443)	2770 (904 to 4890)
Mean of first and second years	2518 (1600 to 3550)	4042 (2832 to 5700)	1524 (−80 to 3404)
Other (eg, helplines, day centers)			
Baseline	3304 (2933 to 3761)	2986 (2669 to 3311)	−317 (−902 to 200)
First year	1638 (1442 to 1853)	1117 (980 to 1251)	−521 (−786 to −290)
Second year	1195 (1048 to 1348)	902 (784 to 1003)	−293 (−512 to −109)
Mean of first and second years	1417 (1276 to 1574)	1009 (896 to 1114)	−407 (−616 to −231)
Police contacts and court appearances			
Baseline	9222 (7571 to 11 062)	7987 (6634 to 9395)	−1235 (−3310 to 996)
First year	6925 (5300 to 8784)	6572 (5529 to 7720)	−352 (−2474 to 1733)
Second year	5871 (4600 to 7210)	4996 (4178 to 5994)	−875 (−2496 to 679)
Mean of first and second years	6398 (5149 to 7782)	5784 (4955 to 6627)	−613 (−2247 to 977)
Incarcerations			
Baseline	2084 (1210 to 3108)	2365 (1556 to 3452)	281 (−940 to 1529)
First year	1675 (1076 to 2334)	2820 (2125 to 3672)	1146 (210 to 2159)
Second year	3064 (2063 to 4192)	3963 (2980 to 5266)	899 (−544 to 2444)
Mean of first and second years	2369 (1666 to 3131)	3392 (2642 to 4284)	1022 (−66 to 2198)
Welfare and disability benefits			
Baseline	2758 (2588 to 2911)	2869 (2761 to 2991)	111 (−94 to 322)
First year	8641 (8233 to 9060)	9154 (8861 to 9489)	512 (−14 to 1053)
Second year	9480 (9049 to 9956)	9574 (9298 to 9863)	94 (−449 to 619)
Mean of first and second years	9061 (8679 to 9507)	9364 (9095 to 9661)	303 (−175 to 823)
Income earned			
Baseline	343 (243 to 454)	146 (83 to 227)	−197 (−329 to −70)
First year	1129 (854 to 1399)	517 (395 to 669)	−611 (−905 to −330)
Second year	1111 (868 to 1375)	863 (671 to 1062)	−248 (−579 to 75)
Mean of first and second years	1120 (906 to 1360)	690 (553 to 835)	−430 (−696 to −141)
Total (excluding intervention cost)			
Baseline	53 015 (48 870 to 56 809)	49 062 (45 971 to 52 654)	−3954 (−8843 to 1479)
First year	44 299 (41 018 to 47 592)	35 372 (33 159 to 37 857)	−8927 (−13 373 to −4895)
Second year	37 398 (34 445 to 40 598)	33 068 (30 612 to 36 188)	−4330 (−8157 to 53)
Mean of first and second years	40 849 (38 374 to 43 538)	34 220 (32 175 to 36 695)	−6629 (−10 199 to −2969)
Total (including intervention cost)			
First year	44 299 (41 018 to 47 592)	50 554 (47 801 to 53 221)	6255 (1617 to 10 425)
Second year	37 398 (34 445 to 40 598)	46 879 (44 243 to 50 135)	9480 (5772 to 13 888)
Mean of first and second years	40 849 (38 374 to 43 538)	48 716 (46 593 to 51 072)	7868 (4409 to 11 405)

Abbreviations: ED, emergency department; HF, Housing First; TAU, treatment as usual.

^a^ Twenty-seven elements (1 per month and 3 before the baseline interview) were included for days of stable housing. Missing rates (addressed using multiple imputation) were 14.8% for the TAU group and 9.4% for the HF group. For costs, 279 elements were included; missing rates were 13.0% and 9.2%, respectively.

^b^ This category includes rooms in buildings with on-site support staff and, notably for Toronto, subsidized rooms in buildings without on-site support staff.

Table 3 disaggregates costs by site rather than by cost category. The magnitude of the net cost, including the cost of the intervention, ranged from $5218 (95% CI, −$983 to $11 385) per person per year in Montreal to $11 702 (95% CI, $5196-$18 873) per person per year in Toronto.

**Table 3.** Total Unadjusted Mean Costs per Person per Year by Site

Cost by Site	TAU Group		HF Group		Difference, Mean Cost (95% CI)
	No. of Participants	Mean Cost (95% CI), Can$	No. of Participants	Mean Cost (95% CI), Can$	
ICM team					
Montreal	NA	NA	200	7375 (6679 to 8075)	NA
Toronto	NA	NA	200	6825 (5850 to 7914)	NA
Winnipeg	NA	NA	177	7613 (6194 to 9148)	NA
Vancouver	NA	NA	100	5533 (4199 to 7211)	NA
Housing team and rent supplements					
Montreal	NA	NA	200	7345 (7026 to 7702)	NA
Toronto	NA	NA	200	8752 (8248 to 9184)	NA
Winnipeg	NA	NA	177	5336 (4903 to 5797)	NA
Vancouver	NA	NA	100	9093 (8310 to 9848)	NA
Total without intervention					
Montreal	102	41 699 (36 752 to 47 040)	200	32 197 (28 598 to 36 031)	−9502 (−16 072 to −3280)
Toronto	159	41 108 (36 436 to 45 935)	200	37 234 (33 096 to 41 897)	−3875 (−10 353 to 3136)
Winnipeg	126	38 961 (34 248 to 43 498)	177	33 987 (30 590 to 37 551)	−4975 (−10 365 to 740)
Vancouver	96	41 992 (35 752 to 48 308)	100	32 653 (27 222 to 40 155)	−9339 (−18 250 to 1008)
Total with intervention					
Montreal	102	41 699 (36 752 to 47 040)	200	46 917 (43 389 to 50 542)	5218 (−983 to 11 385)
Toronto	159	41 108 (36 436 to 45 935)	200	52 811 (48 171 to 57 341)	11 702 (5196 to 18 873)
Winnipeg	126	38 961 (34 248 to 43 498)	177	46 935 (43 045 to 51 066)	7974 (1985 to 14 335)
Vancouver	96	41 992 (35 752 to 48 308)	100	47 279 (41 497 to 55 015)	5287 (−3816 to 15 428)

Abbreviations: HF, Housing First; ICM, Intensive Case Management; NA, not applicable; TAU, treatment as usual.

Days with stable housing were higher by 140.34 (95% CI, 128.14-153.31) days in the HF group, with a cost difference of $7867.73 (95% CI, $4408.81-$11 404.79). Thus, the incremental cost-effectiveness ratio was $56.08 (95% CI, $29.55-$84.78) per day of stable housing.

eFigure 2 in the Supplement shows 500 bootstrap replicates of mean incremental cost and the corresponding mean incremental number of days of stable housing on the cost-effectiveness plane. All the points lie in the quadrant corresponding to higher effectiveness and higher costs, indicating that taking all sites together, the intervention unambiguously increases days of stable housing and costs.

The cost-effectiveness acceptability curve shown in the Figure indicates that if the decision-maker is willing to pay $67 per night of stable housing, there is an 80% chance that HF is cost-effective compared with TAU. If the decision-maker is willing to pay approximately $100 per day of stable housing, then the probability that the intervention is cost-effective increases to 100%.

**Figure.**
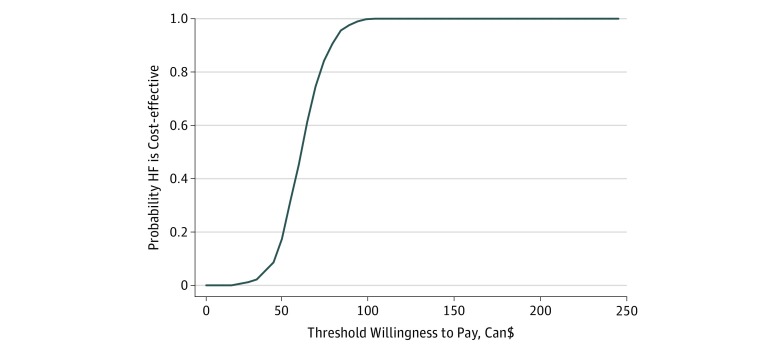
Cost-effectiveness Acceptability Curve The probability that the Housing First (HF) intervention is cost-effective compared with treatment as usual as a function of the value that the decision-maker attributes to an additional day of stable housing.

eTable 1 in the Supplement shows the results of net benefit regressions that do not include interactions. As the decision-maker’s willingness to pay for an additional day of stable housing (represented by λ) rises from $0 to $100, the adjusted net benefit of receiving HF is initially negative (net cost of −$8604 per person per year; 95% CI, −$12 027 to −$5181) but increases quickly so that at $100 the net benefit is positive, reaching $5269 (95% CI, $1352-$9186). Only 1 other variable appeared to be associated with a meaningful difference in net benefit: people who had been arrested or incarcerated in the 6 months before baseline had a lower net benefit. Age, sex, alcohol or substance abuse or dependence, and level of functioning were not associated with net benefit, regardless of λ, after adjusting for site and the other factors.

Table 4 shows the results of adding interactions between group assignment and the variables identified using the procedure described above. None of the site variables or interactions with site were meaningfully different from zero, suggesting that the cost-effectiveness of HF with ICM did not vary by site. Costs appeared to vary with arrest history; the addition of the interaction terms had little effect on estimated coefficients. A higher level of functioning was associated with a higher net benefit at higher levels of λ: at λ = $100, a (clinically meaningful) 10-point increase in the Multnomah Community Ability Scale score was associated with an increase in net benefit of $6901 (95% CI, $1839-$11 962) per person per year, indicating that a higher level of functioning was associated with more days of stable housing. The results suggest that HF yielded a net benefit lower by approximately $7000 for people who had had 2 or more hospitalizations for mental illness during a 1-year period during the previous 5 years; the amount varied from −$6820 (95% CI, −$12 673 to −$967) at λ = 0 to −$7456 (95% CI, −$14 065 to −$847) at λ = 100. None of the other interaction terms appeared to meaningfully alter costs at any value of λ. Thus, no individual-level baseline variable, except possibly hospitalization history, and no site appeared to make HF with ICM more or less cost-effective.

**Table 4.** Net Benefit Regression Results Assigning Different Values to an Additional Day of Stable Housing With Interaction Terms^a^

Term	Estimated β Coefficient (95% CI), 2016 Can$^b^					
	λ^c^ = Can$0	λ^c^ = Can$20	λ^c^ = Can$40	λ^c^ = Can$60	λ^c^ = Can$80	λ^c^ = Can$100
HF intervention	36 433 (−3860 to 76 726)	39 828 (−1193 to 80 850)	43 223 (1304 to 85 143)	46 618 (3643 to 89 594)	50 013 (5834 to 94 193)	53 409 (7890 to 98 927)
Toronto	1088 (−6472 to 8649)	1575 (−6104 to 9254)	2062 (−5770 to 9894)	2548 (−5470 to 10 566)	3035 (−5200 to 11 270)	3522 (−4959 to 12 002)
Winnipeg	1798 (−6198 to 9794)	1453 (−6688 to 9595)	1109 (−7215 to 9432)	764 (−7776 to 9304)	419 (−8370 to 9208)	74 (−8993 to 9142)
Vancouver	1397 (−7020 to 9813)	620 (−7951 to 9192)	−156 (−8920 to 8608)	−932 (−9925 to 8061)	−1708 (−10 964 to 7548)	−2484 (−12 034 to 7065)
Aged 30-49	2833 (−4575 to 10 241)	2781 (−4764 to 10 326)	2729 (−4985 to 10 444)	2677 (−5238 to 10 592)	2625 (−5519 to 10 770)	2574 (−5826 to 10 973)
Aged ≥50 y	431 (−8243 to 9104)	276 (−8571 to 9124)	122 (−8937 to 9181)	−32 (−9339 to 9275)	−186 (−9773 to 9400)	−341 (−10 237 to 9556)
Female	−4015 (−7765 to −265)	−3818 (−7634 to −1)	−3621 (−7520 to 278)	−3424 (−7421 to 573)	−3226 (−7335 to 882)	−3029 (−7263 to 1205)
Alcohol or substance abuse or dependence	427 (−3477 to 4331)	457 (−3508 to 4423)	488 (−3556 to 4532)	519 (−3619 to 4656)	549 (−3697 to 4795)	580 (−3788 to 4947)
MCAS score/10^d^	4859 (377 to 9341)	5267 (705 to 9830)	5676 (1013 to 10 338)	6084 (1304 to 10 864)	6492 (1579 to 11 405)	6901 (1839 to 11 962)
Hospitalization history^e^	−3730 (−7940 to 479)	−3547 (−7834 to 741)	−3363 (−7746 to 1021)	−3179 (−7676 to 1317)	−2996 (−7620 to 1629)	−2812 (−7579 to 1956)
Arrest history^f^	−9895 (−13 836 to −5954)	−10 467 (−14 481 to −6453)	−11 038 (−15 143 to −6934)	−11 610 (−15 820 to −7399)	−12 181 (−16 513 to −7850)	−12 753 (−17 219 to −8287)
Longest period homeless^g^	−13 (−54 to 28)	−16 (−58 to 25)	−19 (−62 to 23)	−22 (−66 to 22)	−25 (−70 to 20)	−28 (−75 to 18)
Interaction terms						
Toronto × HF	−5737 (−15 338 to 3864)	−6198 (−15 966 to 3570)	−6658 (−16 637 to 3321)	−7119 (−17 349 to 3111)	−7579 (−18 099 to 2940)	−8040 (−18 884 to 2804)
Winnipeg × HF	−969 (−11 033 to 9095)	−1755 (−12 008 to 8498)	−2542 (−13 029 to 7946)	−3328 (−14 091 to 7435)	−4114 (−15 192 to 6964)	−4901 (−16 329 to 6528)
Vancouver × HF	−1612 (−12 640 to 9415)	−1218 (−12 457 to 10 021)	−824 (−12 325 to 10 677)	−429 (−12 239 to 11 380)	−35 (−12 196 to 12 126)	359 (−12 192 to 12 911)
Age 30-49 × HF	181 (−9395 to 9756)	683 (−9077 to 10 443)	1185 (-8803 to 11 173)	1687 (−8568 to 11 943)	2190 (−8371 to 12 750)	2692 (−8208 to 13 592)
Age ≥50 × HF	5640 (−5515 to 16 795)	6454 (−4920 to 17 828)	7268 (−4374 to 18 911)	8082 (−3874 to 20 039)	8896 (−3417 to 21 209)	9710 (−2998 to 22 418)
Hospitalization history × HF	−6820 (−12 673 to −967)	−6947 (−12 906 to −989)	−7075 (−13 163 to −986)	−7202 (−13 443 to −960)	−7329 (−13 745 to −914)	−7456 (−14 065 to −847)
Constant	−69 214 (−100 884 to −37 544)	−69 254 (−101 614 to −36 893)	−69 175 (−100 284 to −38 065)	−69 293 (−102 466 to −36 120)	−69 333 (−103 431 to −35 234)	−69 372 (−104 500 to −34 244)

Abbreviations: HF, Housing First intervention; MCAS, Multnomah Community Ability Scale.

^a^ Includes 1160 participants. Models estimated with net monetary benefit are not adjusted for baseline differences in costs. Dependent variable is (d_i_ × λ) – c_i_, where λ is the threshold ratio (in Canadian dollars per additional day of stable housing), d_i_ is participant i’s annualized number of days of stable housing, and c_i_ is the corresponding total cost.

^b^ Reference categories include Montreal site, younger than 30 years, and no alcohol or substance abuse or dependence.

^c^ Decision-maker’s willingness to pay for an additional day of stable housing.

^d^ Coefficients indicate partial association with a 10-point increase in MCAS score.

^e^ Indicates 2 or more hospitalizations for mental illness during a 1-year period within the 5 years before baseline.

^f^ Indicates 1 or more arrests or incarcerations during the 6 months before baseline.

^g^ Indicates during lifetime, in months.

Sensitivity analyses shown in eTable 2 in the Supplement indicate that our results are robust to changes in the discount rate and only somewhat sensitive to the adjustment for baseline differences or a combination of both. Adjusting for baseline differences increases the incremental cost-effectiveness ratio from $56.08 to $60.18. Changes in the discount rate have a minimal effect. The largest change is obtained by adjusting for baseline differences, without altering the discount rate: the incremental cost-effectiveness ratio becomes $60.18 (95% CI, $35.27-$86.95).

## Discussion

This cost-effectiveness analysis relies on data from the largest trial of scattered-site HF with ICM for people with mental illness and moderate needs, to our knowledge, conducted to date. The intervention costs a mean of $14 496 per person per year. Cost offsets on a wide range of other services reduced the net cost to $7868, a 46% reduction. An additional day of stable housing cost $56.08. Cost-effectiveness seemed to be about the same regardless of participant characteristics, with the possible exception of hospitalization history.

In previous reports of the At Home/Chez Soi study,^7,25^ the intervention cost was reported as Can$14 177 per participant annually (in 2011 dollars), and the mean net cost offset was Can $4849, or 34% of the cost of the intervention. Although qualitatively similar, the numerical estimates presented herein differ from the earlier ones for several reasons. Most important, in the present study, we did not adjust for baseline differences in costs, whereas the earlier reports used a relatively simple difference-in-differences method, applied to mean costs per person per site. Second, we allocated the cost of the intervention to individual study participants. Third, we refined several unit cost calculations compared with the earlier report.

Cost-effectiveness analyses, including for the treatment of mental health conditions, often use quality-adjusted life-years as an outcome measure,^19^ despite their known limitations.^26^ In the present report, consistent with the only published cost-effectiveness analysis of a related intervention published to date,^5^ we used days of stable housing instead. Hierarchical linear models estimated on the EQ-5D, a common instrument from which to derive quality-adjusted life-years,^27^ showed no significant difference between the experimental and TAU groups.^7^ This finding is not surprising because HF is in significant measure a social care intervention, with health effects that may only become apparent during a longer period. Qualitative interviews conducted on a 10% sample of study participants showed that participants in the HF group were much more likely to experience positive changes in their life trajectories than those in the TAU group during the 2 years of the study.^28^

The only comparable cost-effectiveness analysis in the literature^5^ was performed at Veterans Affairs medical centers in 4 US cities using data collected in the early to middle 1990s. The most similar intervention in that study was a less intensive form of case management than herein, with as many as 25 participants per case manager. This intervention was combined with Section 8 vouchers (rent subsidies). Fully half of participants in that study^5^ had no psychiatric diagnosis other than alcohol or drug use disorder. The study found that, from a societal perspective, the intervention cost $45 more per additional day of housing, compared with standard care. Adjusting for the effects of inflation^29^ and using purchasing power parity in 2016 to convert US into Canadian dollars,^30^ that amount is equivalent to about Can$79. Our somewhat lower estimate may reflect the greater potential for cost offsets with a group of participants with greater illness.

In this trial, careful attention was paid to implementation fidelity.^3^ Fidelity was found to be fair to excellent across sites.^12^ Other work has reported an association between higher fidelity and housing stability, quality of life, and community functioning^31^ as well as other positive outcomes.^32,33^ Not all HF implementations are as careful to follow the Pathways model as those of the At Home/Chez Soi trial; for instance, caseloads may be increased or rent supplements may be reduced. Although such changes will reduce the cost of the intervention itself, the magnitude of the cost offsets observed herein may not remain the same.

The fact that HF with ICM did not dominate the intervention—that it did not prove more effective and less costly—does not mean that it should not be implemented. Most health and social interventions do not pay for themselves. Rather, they yield benefits judged sufficient to merit their cost. The cost of the intervention itself, which was approximately $40 per participant per day, is well within the range of the costs of many currently funded forms of emergency shelter and supported housing.^13^ Allowing for some budgetary reallocations and reflecting the reductions in costs of these and other services in which the delivery of HF with ICM results, the overall budgetary impact of a significant expansion of HF with ICM capacity is likely to be quite reasonable.

Our study found little evidence of HF being more or less cost-effective for different subgroups. This finding may be attributable to the fact that clinical teams adjust the nature and intensity of their interventions according to each participant’s needs and preferences. Cost-effectiveness may have been lower for people with more previous hospitalizations for mental illness, possibly because the teams expended more effort on clients more prone to prior hospitalizations but could not sufficiently alter participants’ propensity to be hospitalized more often. Our findings give no ground for any selection of participants on characteristics such as current alcohol or substance abuse or previous justice services involvement.

### Strengths and Limitations

Our study presents several strengths. The sample size was large and drawn from several sites. It evaluated a carefully defined and implemented intervention. Attrition, and in particular differential attrition, was modest. Service use was measured in a much more comprehensive way than is typical in cost-effectiveness studies. Unit costs were carefully estimated.

Several limitations need to be noted as well. Service use data were based on participant self-reports. Although these reports are subject to recall biases, the most costly components of costs are based on self-reports gathered at 3-month intervals, which have been shown to have good validity.^34^ The validity of self-reports in the At Home/Chez Soi study has also been corroborated directly.^35,36^ The costs of medications were not included, owing to the difficulty of obtaining reliable and sufficiently detailed information on medications taken by participants by questionnaire and restrictions on sharing of participant-level administrative data, including on medication use, across provinces. The ICM teams, however, do not typically focus on increasing medication adherence. The follow-up period was 2 years, and we do not know how cost-effectiveness would evolve for a longer duration. We did not estimate the administrative costs of transfer payments,^15^ but because most participants in both groups received social assistance payments, doing so would have had little effect.

## Conclusions

In this large, multiple-site trial, we found that about half of the costs of an HF with ICM intervention were offset by reductions in the costs of shelters, ambulatory visits, emergency department visits, and other services. Even if these cost offsets do not result in reductions in budgetary outlays, they translate into greater availability of resources for others in need. The net cost was modest in relation to current expenditures on individuals who are homeless. Furthermore, we found little evidence of cost-effectiveness varying according to participant characteristics. These results support more widespread implementation of scattered-site HF with ICM programs for homeless people with characteristics comparable to those of the participants included in this trial.

## Notes

Supplement. **eFigure 1.** Flowchart Showing Selection of Study Participants **eFigure 2.** Bootstrap Replicates on the Cost-effectiveness Plane **eTable 1.** Net Benefit Regression Results, Assigning Different Values to an Additional Day of Stable Housing Without Interaction Terms **eTable 2.** Two-Way Sensitivity Analysis: ICER and 95% CI as a Function of Discount Rate and Whether an Adjustment Is Made for Baseline Differences
