# Error in eFigure 1 in the Supplement

**DOI:** 10.1001/jamanetworkopen.2019.12622

**Published:** 2019-09-11

In the Original Investigation titled “Cost-effectiveness of Housing First Intervention With Intensive Case Management Compared With Treatment as Usual for Homeless Adults With Mental Illness: Secondary Analysis of a Randomized Clinical Trial,”^1^ published August 21, 2019, there were 2 incorrect numbers in eFigure 1 in the Supplement. The numbers excluded due to missing all Health, Social, and Justice Service Use data or all Residential Time-Line Follow-Back data should be 9 for the intervention arm and 22 for the TAU arm. This article has been corrected.^1^
