# Supplementary material for: Cost-effectiveness of Housing First Intervention With Intensive Case Management Compared With Treatment as Usual for Homeless Adults With Mental Illness: Secondary Analysis of a Randomized Clinical Trial
Source: JAMA Netw Open. 2019 Aug 21;2(8):e199782. doi: 10.1001/jamanetworkopen.2019.9782 (PMC6707012; doi:10.1001/jamanetworkopen.2019.9782)
Supplement: Supplement. — eFigure 1. Flowchart Showing Selection of Study Participants eFigure 2. Bootstrap Replicates on the Cost-effectiveness Plane eTable 1. Net Benefit Regression Results, Assigning Different Values to an Additional Day of Stable Housing Without Interaction Terms eTable 2. Two-Way Sensitivity Analysis: ICER and 95% CI as a Function of Discount Rate and Whether an Adjustment Is Made for Baseline Differences [file jamanetwopen-2-e199782-s001.pdf]

Supplementary Online Content

Latimer EA, Rabouin D, Cao Z, et al; At Home/Chez Soi Investigators. Cost-effectiveness of Housing First intervention with Intensive Case Management compared with treatment as usual for homeless adults with mental illness: secondary analysis of a randomized clinical trial. *JAMA Netw Open*. 2019;2(8):e199782. doi:10.1001/jamanetworkopen.2019.9782

eFigure 1. Flowchart Showing Selection of Study Participants

eFigure 2. Bootstrap Replicates on the Cost-effectiveness Plane

eTable 1. Net Benefit Regression Results, Assigning Different Values to an Additional Day of Stable Housing Without Interaction Terms

eTable 2. Two-Way Sensitivity Analysis: ICER and 95% CI as a Function of Discount Rate and Whether an Adjustment Is Made for Baseline Differences

This supplementary material has been provided by the authors to give readers additional information about their work.

© 2019 Latimer EA et al. *JAMA Network Open*

eFigure 1. Flowchart Showing Selection of Study Participants

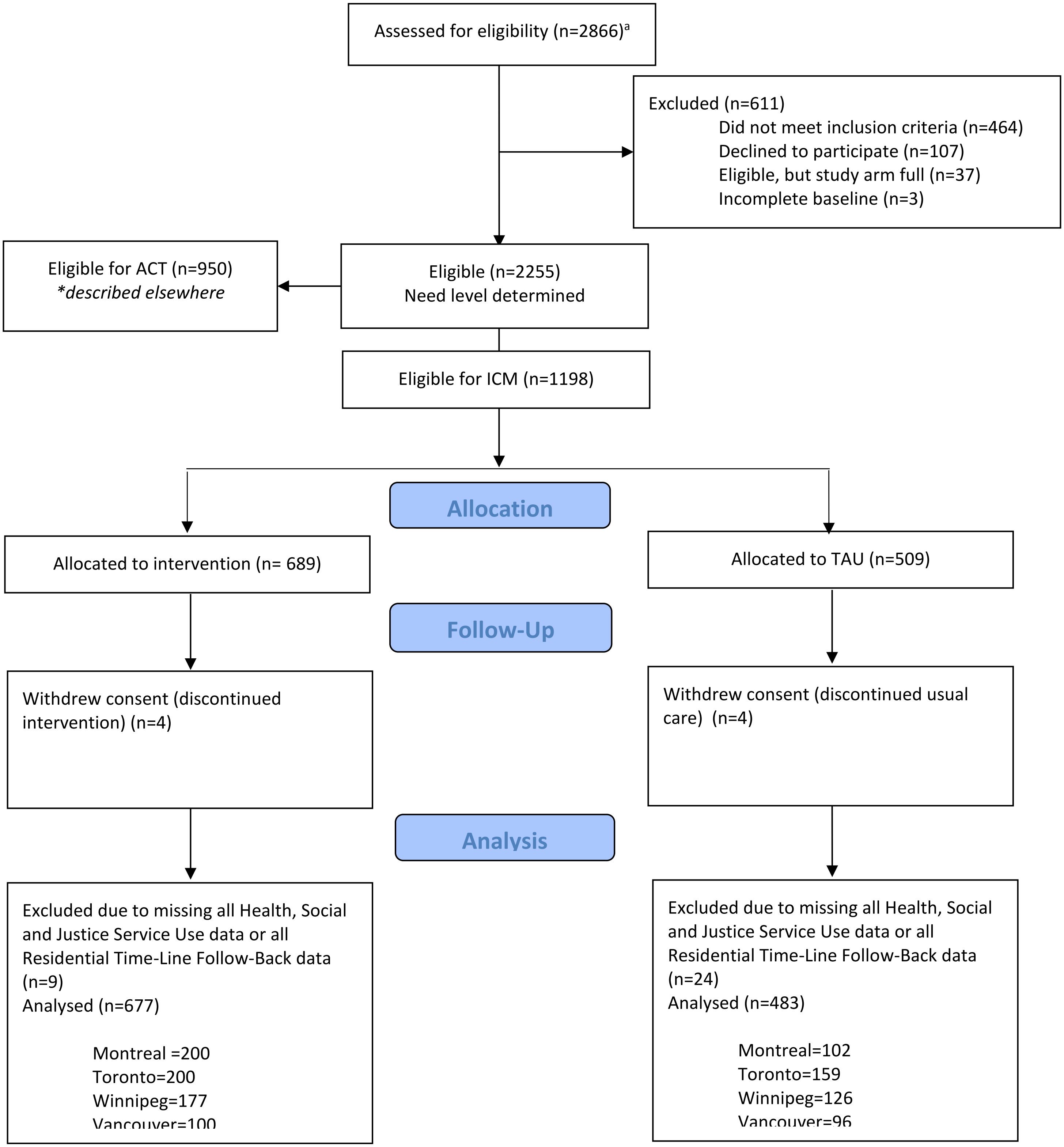

Abbreviations: ACT, Assertive Community Treatment; ICM, Intensive Case Management; TAU, Treatment as usual.
[a]The number of participants assessed for eligibility is an estimate as some sites employed pre-screening and did not document those who were excluded through this process.

© 2019 Latimer EA et al. *JAMA Network Open*

eFigure 2. Bootstrap Replicates on the Cost-effectiveness Plane

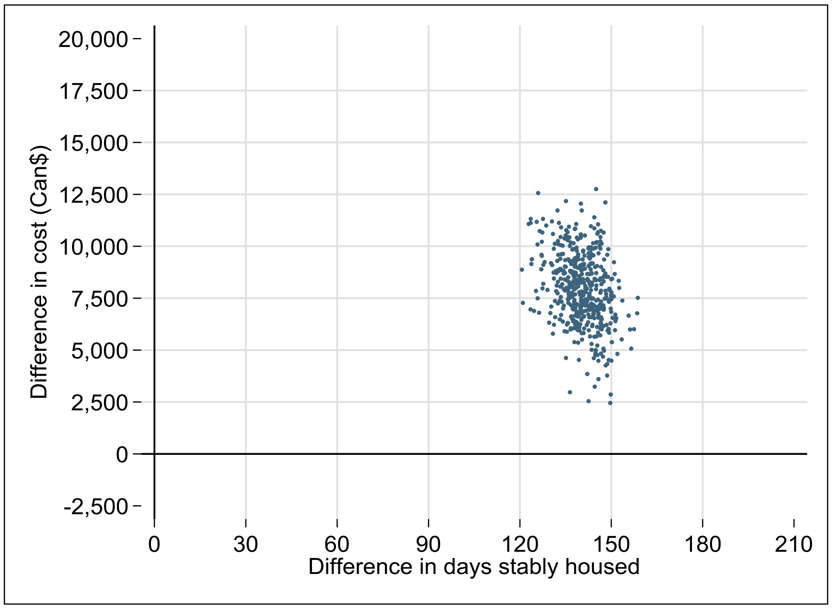

The original number of days stably housed and the total cost for each Housing First and Treatment as Usual participant are used to calculate the difference in average number of days stably housed between the Housing First and Treatment as Usual groups (Housing First minus Treatment as Usual), and the difference in average total costs. These two differences can be plotted on the cost-effectiveness plane; their ratio is the incremental cost-effectiveness ratio. To assess the variability in that ratio, 500 bootstrap samples are drawn. (A bootstrap sample is randomly drawn with replacement from the original set of cost-effect pairs, so that it has the same number of experimental and control group cost-effect pairs as in the original sample.) For each bootstrap sample, the difference in days stably housed and in total costs is computed and plotted. The cost-effectiveness acceptability curve (Figure 1) can then be derived from this figure.

© 2019 Latimer EA et al. *JAMA Network Open*

eTable 1. Net Benefit Regression Results, Assigning Different Values to an Additional Day of Stable Housing Without Interaction Terms[a b]

	λ[c]=$0			λ=$20			λ=$40		
	b	95% C.I.		b	95% C.I.		b	95% C.I.	
HF	-8,604	-12,027	-5,181	-5,830	-9,321	-2,338	-3,055	-6,631	522
Toronto[d]	-2,361	-7,085	2,363	-2,174	-6,977	2,629	-1,987	-6,891	2,916
Winnipeg[d]	1,198	-3,793	6,189	375	-4,705	5,454	-449	-5,640	4,743
Vancouver[d]	242	-5,332	5,815	-382	-6,068	5,304	-1,006	-6,829	4,816
Age 30-49[e]	2,941	-1,877	7,759	3,158	-1,743	8,059	3,374	-1,631	8,380
Age 50+[e]	3,996	-1,655	9,647	4,304	-1,454	10,062	4,612	-1,278	10,502
Female	-3,871	-7,617	-124	-3,682	-7,495	132	-3,492	-7,389	405
Alcohol or Substance abuse or dependence[f]	647	-3,265	4,559	685	-3,290	4,659	722	-3,331	4,776
MCAS score/10[g]	918	-1,910	3,746	1,260	-1,622	4,142	1,602	-1,346	4,550
Hospitalization history[h]	-3,639	-7,843	565	-3,447	-7,730	836	-3,255	-7,635	1,125
Arrest history[i]	-9,748	-13,678	-5,818	-10,334	-14,338	-6,330	-10,920	-15,015	-6,824
Longest period homeless[j]	-12	-53	29	-15	-57	27	-18	-61	25
Constant	-43,338	-63,403	-23,273	-43,024	-63,470	-22,578	-42,709	-63,624	-21,795
	λ=$60			λ=$80			λ=$100		
HF	-280	-3,957	3,396	2,494	-1,296	6,285	5,269	1,352	9,186
Toronto	-1,801	-6,824	3,223	-1,614	-6,776	3,549	-1,427	-6,745	3,891
Winnipeg	-1,272	-6,597	4,053	-2,095	-7,573	3,383	-2,918	-8,567	2,731
Vancouver	-1,630	-7,612	4,352	-2,254	-8,416	3,909	-2,877	-9,239	3,484
Age 30-49	3,591	-1,539	8,721	3,807	-1,466	9,081	4,024	-1,411	9,459
Age 50+	4,919	-1,126	10,965	5,227	-995	11,449	5,535	-884	11,953
Female	-3,303	-7,299	693	-3,114	-7,222	995	-2,924	-7,159	1,310
Alcohol or Substance abuse or dependence	760	-3,389	4,909	798	-3,460	5,056	835	-3,545	5,216
MCAS score/10	1,944	-1,081	4,969	2,286	-827	5,400	2,629	-583	5,840
Hospitalization history	-3,063	-7,557	1,431	-2,871	-7,495	1,752	-2,679	-7,446	2,088
Arrest history	-11,506	-15,708	-7,303	-12,092	-16,416	-7,767	-12,678	-17,138	-8,217
Longest period homeless	-21	-65	23	-24	-69	21	-27	-73	20
Constant	-42,395	-63,861	-20,930	-42,081	-64,173	-19,989	-41,767	-64,554	-18,979

© 2019 Latimer EA et al. *JAMA Network Open*

Abbreviations: C.I., confidence interval; MCAS, Multnomah Community Ability Scale.

Notes: [a] N=1160; data are given in 2016 Canadian dollars; [b] Models estimated with net monetary benefit not adjusted for baseline differences in costs – dependent variable is $(d_i \cdot \lambda) - c_i$, where d_i is participant i's annualized number of days stably housed and c_i the corresponding total cost; [c] Decision-maker's willingness to pay for an additional day stably housed; [d] Montreal is the reference category; [e] Age less than 30 is the reference category; [f] No alcohol or substance abuse or dependence is the reference category; [g] Coefficients indicate partial association with a 10-point increase in MCAS score; [h] 2 or more hospitalizations for mental illness over a one-year period during the five years before baseline; [i] one or more arrests or incarcerations in the 6 months before baseline; [j] Over lifetime, in months.

© 2019 Latimer EA et al. *JAMA Network Open*

eTable 2. Two-Way Sensitivity Analysis: ICER and 95% CI as a Function of Discount Rate and Whether an Adjustment is Made for Baseline Differences

	Discount rate		
	**0%**	**3%**	**5%**
**No adjustment for baseline differences**	$55.84 (30.25, 84.59)	$56.08 (29.55, 84.78)	$55.41 (29.95, 84.09)
**Adjustment for baseline differences**	$59.67 (34.61, 87.08)	$60.18 (35.27, 86.95)	$59.27 (34.36, 85.88)

© 2019 Latimer EA et al. *JAMA Network Open*
